# Age, mode of conception, health service use and pregnancy health: a prospective cohort study of Australian women

**DOI:** 10.1186/1471-2393-13-88

**Published:** 2013-04-08

**Authors:** Jane Fisher, Karen Wynter, Karin Hammarberg, John McBain, Frances Gibson, Jacky Boivin, Catherine McMahon

**Affiliations:** 1 Jean Hailes Research Unit, School of Public Health and Preventive Medicine, Monash University, Clayton, , Victoria, 3168, Australia; 2 School of Population Health, University of Melbourne, Carlton, Victoria, 3010, Australia; 3 Melbourne IVF, 320 Victoria Parade, East Melbourne, Victoria, 3002, Australia; 4 Institute of Early Childhood, Macquarie University, Sydney, NSW 2109, Australia; 5 School of Psychology, Cardiff University, Cardiff, CF10 3AT, UK; 6 Centre for Emotional Health, Department of Psychology, Macquarie University, Sydney, NSW 2109, Australia

## Abstract

**Background:**

There is limited evidence about the ways in which maternal age and mode of conception interact with psychological, sociodemographic, health and health service factors in governing pregnancy health. The aim of this study was to establish in what ways maternal age and mode of conception are associated with, health behaviours, health service use and self-rated physical and mental health during pregnancy.

**Method:**

A prospective cohort study was conducted in a collaboration between universities, infertility treatment services and public and private obstetric hospitals in Melbourne and Sydney, Australia,. Consecutive cohorts of nulliparous English-literate women at least 28 weeks pregnant who had conceived through ART (ARTC) or spontaneously (SC) in three age-groups: 20–30; 31–36 and at least 37 years were recruited. Data were obtained via structured individual telephone interviews and self-report postal questionnaires at recruitment and four months postpartum. Study-specific questions assessed: sociodemographic characteristics; reproductive health; health behaviours and health service use. Standardized instruments assessed physical health: SF 12 Physical Component Score (PCS) and mental health: SF12 Mental Component Score (MCS); State Trait Anxiety Inventory and Edinburgh Postnatal Depression Scale. The main outcome measures were the SF 12 PCS, SF12 MCS scores and pregnancy-related hospital admissions.

**Results:**

Of 1179 eligible women 791 (67%) participated, 27 had fertility treatment without oocyte retrieval and were excluded and 592/764 (78%) completed all pregnancy assessments. When other factors were controlled speaking a language other than English, having private health insurance and multiple gestation were associated with worse physical health and having private health insurance and better physical health were associated with better mental health. Pregnancy-related hospital admissions were associated with worse physical health and multiple gestation.

**Conclusions:**

Maternal age and mode of conception are not associated with pregnancy health and health service use when sociodemographic factors are considered.

## Background

In most of the world’s high income countries, as women have attained access to reliable contraception and greater equality of participation in post-secondary education and employment, there has been a consistent increase in the average age at which they have married and given birth. National data in these settings are reported as total fertility rates which are calculated as the average number of babies a woman could expect to bear during her reproductive lifetime, if current age and country-specific fertility rates were experienced. In Australia in the last four decades total fertility rates among women aged less than 30 years have fallen and those among older women have increased. Since 2003 the fertility rate for women aged 35–39 years has exceeded that of women aged 20–24 years [1]. In England and Wales the average age at which women first gave birth in 2009 was 27.6 years and more than 47% of births were to women aged at least 30 years [2]. In the United States of America the average age of first birth among women increased from 21.4 years in 1970 to 25.0 years in 2006 [3]. In these settings there has also been an increase in the proportion of births following conception by assisted reproductive technologies (ART). In England four times more women gave birth following ART conception in 2006 than had done so in 1992 [4]. In Australia 3.2% of births followed assisted conception in 2008 [5] compared with 1.5% of births in 1998 [6].

While definitions of older maternal age vary, there is general agreement that perinatal health risks are more prevalent amongst older than younger women [7]. Joseph et al. [8] analysed data about the 157,445 births in Nova Scotia, Canada between 1988 and 1995. They found that compared to women aged 20 – 24 years, those aged 35–39 years were more likely to experience hypertension (ARR 2.32, 95% CI 1.97 – 2.72); diabetes mellitus (ARR 2.85, 95% CI 1.89 – 4.28); placental abruption (ARR 1.64, 95% CI 1.24 – 2.16) and a coincidental chronic disease (ARR1.90, 95% CI 1.66 – 2.18). Similarly Jolly et al. [9] investigated 385,120 pregnancies in North West Thames Region, England and compared outcomes in women aged 18 – 34 years with those of women aged at least 35 years. Rates of gestational diabetes (OR 2.63, 99% CI 2.40 – 2.89); placenta praevia (OR 1.93, 99% CI1.58 – 2.35) and breech presentation (OR 1.37, 99% CI1.28 – 1.47) were higher among the older than the younger women.

Ectopic pregnancy rates increase with age probably because of increased exposure to pelvic inflammatory disease including unrecognized and untreated infections and the associated tubal occlusions [10] Spontaneous abortions are more common among older than younger women attributed in part to increased rates of chromosomal abnormalities among them [10]. Even when gestational age, ethnicity, previous vaginal birth and foetal presentation are controlled, older women are more likely than younger women to have a caesarean birth. This is suggested to be because they have less compliance in the pelvic joints and reduced oxytocin receptors which govern onset and progress of labour [11] but it might also be because of more cautious obstetric management [12]. Pre-eclampsia and chronic hypertension are more common in older than younger pregnant women [11,13]. Maternal mortality, while rare in high-income countries, is twice as prevalent among women aged older than 35 than those younger than 30 years attributed in particular to pulmonary embolism, amniotic fluid embolism and hypertension [7].

While not the only cause of fertility difficulties, as a result of depletion in follicular reserves and a reduction in uterine receptivity, fertility decreases with age in women [14,15]. Where services are available people experiencing difficulties in conceiving can seek infertility treatment with assisted reproductive technologies (ART). However, these are expensive, and can be experienced as intrusive, and their use does not assure pregnancy. Overall couples who are more socioeconomically advantaged, in which the woman is older and nulliparous are the group most likely to seek treatment [16]. Compared to pregnancy after spontaneous conception, pregnancy after conception with in vitro fertilization is associated with increased risks of gestational diabetes (RR = 2); gestational hypertension (RR = 1.6); placenta praevia (RR = 2.9) and caesarean birth (RR range = 1.5 – 2.1). Perinatal outcomes are also worse, with increased risks of pre-term birth (RR range 1.4 – 2.0); very low birth weight (RR range 1.8 – 3.0) and perinatal mortality (RR = 1.7) [17].

There is emerging evidence that pregnancy mental health and psychological functioning also vary with age and mode of conception. Koleva et al. [18] examined risk factors including maternal age for depressive symptoms in 5404 pregnant women recruited systematically in Iowa, USA. They found that symptoms of depression were most prevalent among young, less well educated, unmarried women with unintended pregnancies who were living on low incomes. Similarly Rich-Edwards et al. [19] found in 1662 pregnant American women participating in a cohort study that risk of depressive symptoms was highest among young women, and suggested this was attributable to financial difficulties, being single and having an unwanted pregnancy. Lampinen et al. [20] reviewed descriptors of risk in obstetric literature and concluded that the focus on risk associated with pregnancy at an older than average age is likely to lead to increased anxiety about maternal and foetal health among older women. However, in a comparison of small groups Robb et al. [21] found self reported stress was no more prevalent among women aged 35 or older than those aged 20 – 30 years. In a systematic review Hammarberg et al. [22] concluded that women who conceive with ART are found consistently to have lower rates of symptoms of depression and anxiety during pregnancy than women who conceive spontaneously. McMahon et al. [23] found in a cohort of women conceiving with ART that those aged older than 37 years were no more likely to have pregnancy-specific anxiety than those younger than 35 years.

In general these comparisons of pregnancy-related risks do not take into account the potential confounding effects of socioeconomic circumstances, protective health behaviours and access to and use of health services. Older women are more likely to have established an occupational identity, be employed, partnered and economically secure than younger women, which might enable them to have greater choice in health care and to afford to participate in health promoting activities. Klemetti et al. [24] examined self-reported health, pregnancy symptoms and antenatal health care use in 4800 women randomly selected by the Office of National Statistics in England from all those giving birth in one week in 2006. They found that women aged over 35 had less vomiting (AOR 0.49, 95% CI0.35 – 0.70); backache (AOR 0.42, 95% CI 0.32 – 0.55); and depression (AOR 0.58, (5% CI 0.37 – 0.90) than women aged less than 25 years, but were more likely to have hemorrhoids (AOR 1.81, 95% CI 1.31 – 2.47). The older women had fewer antenatal hospital admissions and, with the exception of screening for Down Syndrome, fewer pregnancy health care consultations than younger women. In Aberdeen Scotland, Bell et al. [12] examined perinatal data for 28,484 women who gave birth to a singleton infant 1988 – 1997. They found that rates of interventions increased with age, but were not explained completely by obstetric complications, suggesting that perceptions of risk govern health service use and health service provision.

The Parental Age and Transition to Parenthood Australia (PATPA) study is an investigation of the interactions among maternal age and mode of conception in governing the transition to parenthood taking psychological, sociodemographic, health and health service factors into account. The aim of this part of the PATPA study was to establish in what ways maternal age and mode of conception are associated with self-rated physical and mental health, health service use and health behaviours during pregnancy.

## Methods

The PATPA study used a prospective cohort design with assessments in late pregnancy and four months postpartum of a sample stratified by age and by mode of conception. The methods have already been reported (McMahon et al., 2011), but are summarized briefly here.

### Setting

The study was undertaken in Australia which has a two-tier health system in which there are state provided medical and hospital services accessible to all citizens, who can elect to purchase private health insurance which enables them to receive treatment in private hospitals. It was a collaboration between academic institutions (the University of Melbourne and Macquarie University), infertility treatment services (Melbourne IVF and IVF Australia) and public and private hospitals providing obstetric health care to women in the capital cities of two Australian states: Melbourne in Victoria and Sydney in New South Wales.

### Participants and recruitment

To address our primary objective of disaggregating the effects of age and mode of conception the sampling strategy was to recruit equal-sized consecutive cohorts of women who had conceived through ART (ARTC) and spontaneously (SC) in three age-groups. The younger group was women aged 20–30 years; the middle group was aged 31–36 years and the older group was aged at least 37 years. Inclusion criteria were to have adequate English fluency to complete questionnaires, and be nulliparous and at least 28 weeks pregnant. Potential SC participants were informed of the study by research assistants at antenatal clinics in public hospitals or antenatal education classes at private hospitals. Potential ARTC participants were informed of the study by the fertility clinic at the time of ultrasound confirmation of pregnancy. Those who agreed to consider participation gave their contact details and were approached four months later to indicate whether or not they consented to join the study. A priori power calculations were based on group differences between age groups. Power calculations using G*Power (Buchner et al., 2007) using regression effect size and 18 potential predictors (effect size f^2^=0.15, α = 0.05, power =0.95) indicated that a minimum sample of 213 was required in each mode of conception group. Recruitment continued until at least 80 participants (71 + 9 to allow for 10% attrition) had been recruited to each of the six age-group by mode of conception cells.

### Data sources

Data were obtained via both study-specific and standardized instruments administered as structured individual telephone interviews and as self-report postal questionnaires at recruitment and four months postpartum. In the antenatal questionnaire questions were asked about experiences in the first two trimesters of pregnancy and in the postnatal questionnaire about the last trimester of pregnancy.

#### Sociodemographic characteristics

The sociodemographic characteristics which were assessed by study-specific questions included women’s: age, country of birth, language spoken at home, marital, educational and occupational status and whether or not they held private health insurance.

#### Reproductive health

Reproductive health was assessed in study-specific questions about time to pregnancy, mode of conception, whether pregnant with a single or multiple fetuses and, for ARTC participants, whether or not donated gametes had been used.

#### Self-reported physical health

Self-reported physical health was assessed used the Physical Component Summary (PCS) score from the SF-12 [25]. The SF-12 is a 12-item tool developed from the original Short Form–36 Health Survey [26]. It is a self-report measure used for assessing health-related quality of life, which has been shown to be psychometrically robust [27]. It assesses eight physical and mental health dimensions: physical functioning, role limitations due to physical health problems, role limitations due to emotional health problems, social functioning, emotional well-being, pain, energy and/or fatigue, and general health. From these eight dimensions, Physical Component Summary (PCS) is calculated by algorithm. High scores indicate more optimal self-reported subjective health functioning.

Women were also asked to rate how they had been feeling physically overall throughout the pregnancy using a single question with 5 fixed response options, and to select from a list of common symptoms and complications any they had experienced during the pregnancy.

#### Self-reported mental health

Valid, reliable standardized psychometric instruments with established sensitivity to symptoms of depression and anxiety and general emotional wellbeing in ARTC and SC pregnant women [23,28,29] were used.

These included the State-Trait Anxiety Inventory (STAI) [30] which comprises two 20-item scales assesses current (state) and general (trait) experiences of anxiety symptoms. Item scores range from 1 to 4 with higher total scores indicating greater anxiety. Cronbach’s alpha co-efficients in this sample were 0.86 for the state and 0.91 for the trait scales [29]. A cut-off of >40 is recommended to determine cases of anxiety in the third trimester of pregnancy [31]. The Edinburgh Postnatal Depression Scale [32] is a 10-item self-report scale to detect symptoms of depression. Scores for each item range from 0 to 3, yielding a total score between 0 and 30. Higher scores indicate more depressive symptoms [32]. The measure has been validated for use in pregnancy [33]. In pregnancy, the measure is referred to as the Edinburgh Depression Scale (EDS) [34]. Cronbach’s alpha co-efficient in this cohort was 0.86 [29]. The recommended clinical cut-off point for probable major depression in English speaking women is 13 or more postnatally and 15 or more in pregnancy [34]. The Mental Component Summary (MCS) of the SF-12 [25] calculated by algorithm provides a self-assessment of overall emotional wellbeing. High scores indicate more optimal health.

Women were also asked to rate how they had been feeling emotionally overall throughout the pregnancy using a single question with 5 fixed response options and whether they had experienced anxiety or depression during the pregnancy.

#### Health behaviours

The health behaviours that were assessed included: use of prescribed and over-the-counter medications for mental health problems, sleep hypertension, fatigue, gastrointestinal symptoms, headache and backache; use of tobacco and alcohol during pregnancy; and participation in walking, swimming or cycling; gym, aerobics or other vigorous exercise; yoga, thai chi or less vigorous exercise; and competitive sport.

#### Health service use

Study-specific items were used to assess which antenatal health care providers and other medical or allied healthcare providers and complementary practitioners had been consulted during the pregnancy. Women were asked about use of antenatal checks including ultrasound scans and genetic screening and diagnostic testing, any hospital admissions, and to make an overall rating of antenatal care.

### Ethics

Ethics approval and permission to transfer data across the state border were obtained from the human research and ethics committees of Macquarie University, the University of Melbourne, IVF Australia, Melbourne IVF, South East Sydney and Illawarra Area Health Service, Northern Sydney and Central Coast Area Health Service, the Royal Women’s Hospital and Frances Perry House.

### Procedure

Participation involved completion of a computerized telephone interview and a set of self-report questionnaires at the point of recruitment in the third trimester of pregnancy. Follow up interviews and questionnaires were completed at four months after the birth; the postnatal questionnaires included questions about physical symptoms and complications, as well as hospital admissions, in the final trimester of pregnancy.

### Data management and analysis

All data were entered into password protected computer files. Data about pregnancy health were drawn from both the antenatal and postnatal assessments and combined when the information was collected at both time points.

Distributions of continuous variables were checked for normality, skewness and kurtosis. On the basis of distribution of responses categorical variables were recoded into binary variables for multivariable analyses. Responses to overall appraisals on five-item Likert scales of how women had felt physically and emotionally during pregnancy were recoded into binary factors: *I have been feeling very well* versus all other responses and *I have been feeling very or mostly calm and relaxed* versus all other responses, respectively. If respondents indicated that they had been admitted to hospital at any stage during pregnancy, we examined their text responses giving reasons for hospitalisation and were then able to create a binary variable: any pregnancy-related hospitalisation or none.

Three binary variables were created indicating any or no participation in any form of exercise at least once a week, smoking or alcohol use during pregnancy. Responses to the question *Overall, how would you describe your antenatal care* were recoded into *very good* or g*ood* versus all other responses.

Univariate associations between self-reported health, health behaviours and health service use, and age and mode of conception groups were calculated using chi-squared tests for comparisons between categorical variables, t-tests or ANOVA for normally distributed continuous variables, and non-parametric Mann–Whitney or Kruskal-Wallis tests for ordinal or non-normally distributed continuous variables. In the case of non-normal distributions although non-parametric tests were used to test for between-group differences, we report means for ease of interpretation. As access is governed by socioeconomic status, use of health services was examined separately for women with and without private health insurance.

The outcomes were SF12 PCS and MCS scores and pregnancy-related antenatal admissions. In order to determine which independent variables to include in the multivariable models, we tested for significant univariate associations between the outcome variables and socio-demographic and reproductive history; self-rating of physical and emotional health; and scores on the EDS and State Trait Anxiety Inventory. Factors which were associated significantly with the outcome variables, and socio-demographic factors which were significantly different between SC and ARTC groups were entered into hierarchical regression models (PCS and MCS scores] and a logistic regression model (any hospital admissions during pregnancy].

## Results

A total of 1179 women met eligibility criteria and were approached of whom 791 (67%] agreed to participate. From ART recruitment sites, 329/542 (61%] of women consented while 462/637 (73%] of women consented from other recruitment sites. After recruitment, 27 women (8 aged 20–30 years; 10 aged 31–36 years and 9 aged 37 years] reported use of fertility treatments which did not involve oocyte retrieval (ovulation induction or artificial insemination) and were excluded as they did not meet criteria for being in either the ARTC or SC groups. Of the remaining 764 participants, 592 (78%) completed both interview and questionnaire measures during the third trimester of pregnancy. Complete pregnancy data was provided by more ARTC (297/392; 90%) than SC participants (295/435; 68%). The 98 women who were interviewed in pregnancy and subsequently did not return questionnaires were significantly younger (p<0.001) and less socio-economically advantaged (less likely to have private health insurance and a post-secondary educational qualification, p<0.001). The final sample comprised 178 women in the younger age group, 218 in the middle age group and 196 in the older age group. The mean gestational age at interview was 31.6 weeks, SD = 2.5 weeks, range = 25–40 weeks.

### Sociodemographic characteristics and reproductive health

As is usual in self-report surveys, the participants in this study were relatively socio-economically advantaged, an effect that was amplified by the intentional over-sampling of ARTC women who are more likely than other women of reproductive age in Australia to hold private health insurance [35]. Most participants in this study had private health insurance, post-secondary education and were in professional occupations and married (see Table 1). All ARTC women had experienced oocyte retrieval and fresh or frozen embryo transfer after IVF or ICSI. Some had also used donor sperm [18], donor eggs [9] or donor embryos [2]. ARTC women were older than SC women, and were more likely to have been born in Australia, have private health insurance, have had previous pregnancies, to be expecting multiples, and to be “very pleased” about the current pregnancy. They were significantly less likely to be in paid employment (part- or full-time) and their time to pregnancy was significantly longer than SC women.

**Table 1:** Sociodemographic characteristics and reproductive health of 592 nulliparous pregnant women recruited systematically in Melbourne and Sydney, Australia

	**Total**	**SC**	**ARTC**
		**n=295**	**n=297**
Age Mean (SD)	33.71 (4.92)	32.05 (4.65)	35.37 (4.62)***
Born in Australia n (%)	425 (71.8)	196 (66.4)	229 (77.1)**
Only English spoken at home n (%)	493 (83.3)	238 (80.7)	255 (85.9)
Married n (%)	464 (78.4)	220 (74.6)	244 (82.2)*
Post-secondary education n (%)	486 (82.1)	251 (85.1)	235 (79.1)
Professional occupation n (%)	442 (74.7)	212 (72.4)	230 (78.0)
In paid employment (part- or full-time) n (%)	450 (76.0)	243 (82.4)	207 (69.7)***
Have private health insurance n (%)	466 (78.7)	205 (69.5)	261 (87.9)***
Have had previous pregnancy/ies n (%)	222 (37.5)	94 (31.9)	128 (43.1)**
Previous miscarriage n (%)	154 (26.0)	55 (18.6)	99 (33.3)
Previous termination n (%)	86 (14.5)	46 (15.6)	40 (13.5)
Previous stillbirth / neonatal death n (%)	3 (0.5)	1 (0.3)	2 (0.7)
Time to pregnancy Mean (SD) (months)	24.45 (62.83)	9.73 (59.64)	39.08 (62.59)***
Expecting multiples n (%)	28 (4.7)	5 (1.7)	23 (7.7)***
Feelings about current pregnancy n (%):			
Not pleased	17 (2.9)	16 (5.4)	1 (0.3)
Pleased, but it was a bit inconvenient	41 (6.9)	39 (13.3)	2 (0.7)
Very pleased	533 (90.2)	239 (81.3)	294 (99.0)***

* p<0.05; ** p<0.01; *** p<0.001.

### Pregnancy physical and mental health, health behaviours and health service use

Data about self-reported physical and mental health, health behaviours and health service use by age and mode of conception are presented in Table 2 and 3.

**Table 2:** Health and health behaviours by age group and mode of conception (% unless otherwise specified)

	**Age group (years)**			**Mode of conception**	
	**Up to 30**	**31 – 36**	**37 and older**	**SC**	**ARTC**
	**n = 178**	**n = 218**	**n = 196**	**n = 295**	**n = 297**
**Physical health**					
SF12 Physical Component Score (PCS) Mean (SD)^1^	43.9 (8.0)	43.4 (9.3)	43.5 (8.5)	44.1 (8.2)	43.1 (9.1)
Self-rated general health Mean (SD) 1=Poor to 5=Excellent^1^	4.0 (0.8)	4.1 (0.8)	4.1 (0.8)	4.0 (0.8)	4.1 (0.8)
Feeling very well during pregnancy	28.1	**28.0**	37.2*	28.1	34.0
**Health problems in first two trimesters:**					
Nausea / vomiting	84.3	77.5	76.0	80.7	77.4
Stiff or painful joints	58.4	58.1	49.2	**59.5**	51.0*
Tender breasts	85.3	83.4	79.6	**87.8**	77.7*
Vaginal discharge or irritation	56.7	56.9	53.4	56.9	54.4
Heart palpitations	**19.7**	34.6**	29.9*	28.2	28.8
**Health problems in third trimester**^**2**^					
Carpal Tunnel syndrome	13.9	18.2	19.0	17.7	16.7
Gestational diabetes	4.6	4.2	9.0	**3.2**	8.5**
Pelvic instability	18.5	24.8	21.7	23.4	20.4
Pre-eclampsia	**2.9**	7.0	8.5*	5.0	7.5
Placenta praevia	**1.2**	5.1*	5.3*	**2.1**	5.8*
Baby growing less well than expected	4.6	6.1	6.9	6.4	5.4
Premature labour	7.5	7.0	7.9	7.1	7.8
**Health problems during pregnancy**^**3**^					
High blood pressure	12.9	11.0	12.2	10.8	13.1
Severe Tiredness	87.6	86.7	88.3	88.5	86.5
Haemorrhoids /other bowel problems	44.9	55.5	55.6	51.2	53.5
Indigestion / heartburn	83.7	80.3	81.6	81.4	82.2
Headaches / migraines	69.7	63.3	64.8	66.4	65.0
Back pain	83.7	81.2	76.5	**83.7**	77.1*
Vaginal bleeding	24.2	21.6	30.1	**21.4**	29.0*
**Mental health**					
SF12 Mental Component Score (MCS) Mean (SD)^1^	52.9 (7.3)	53.1 (8.2)	54.1 (8.2)	52.7 (7.9)	54.0 (8.0)
Episodes of intense anxiety in first two trimesters	49 (27.5)	74 (34.1)	55 (28.4)	94 (32.0)	84 (28.5)
Anxiety or depression in third trimester	7.5	8.4	7.4	9.9	5.8
EDS total score Mean (SD)^1^	**6.0 (4.5)**	5.1 (4.3)	4.6 (4.4)**	**5.8 (4.6)**	4.6 (4.2 )***
EDS Score >14	4.5	3.2	3.1	4.8	2.4
STAI A-State total score Mean (SD)^1^	32.4 (8.2)	31.6 (9.0)	30.8 (8.6)	**32.7 (9.6)**	30.5 (7.4)*
STAI state score >40	16.4	14.3	14.8	17.7	12.5
STAI A-Trait total score Mean (SD)^1^	**34.8 (8.3)**	34.0 (8.7)	32.8 (8.0)*	**34.7 (8.6)**	33.0 (8.1)*
STAI trait score >40	24.7	21.7	17.3	21.8	20.5
Mostly or very calm and relaxed	**52.8**	63.8*	69.9**	59.0	66.0
**Health behaviours during pregnancy**					
Used any prescription medication	13.5*	14.7*	**23.0**	15.9	18.2
Used any over-the-counter medication	42.1	46.8	46.4	**40.7**	49.8*
Any smoking	2.8	3.7	2.6	4.1	2.0
Any alcohol	14.7	20.3	16.0	18.1	16.3
Exercising at least weekly	94.9	**97.7**	90.8**	96.3	92.9
**Hospital admissions**^**3**^					
Pregnancy-related	13.5	15.1	18.4	**11.5**	19.9**
Non pregnancy-related	0.6	2.3	3.1	**0.3**	3.7**

^1^ For ease of interpretation, means are reported. However, non-parametric Mann–Whitney tests were used to test for between group differences, as the distribution was non-normal.

^2^ Derived from postnatal questionnaires items assessing final trimester pregnancy health retrospectively, n=576.

^3^ Derived from pregnancy and postnatal questionnaires.

* p<0.05; ** p<0.01; *** p<0.001.

Bold indicates statistically significantly different groups.

**Table 3:** Health service use by age group and mode of conception (% unless otherwise specified), for women with and without private health insurance

	**No private health insurance**					**Private health insurance**				
	**Age group (years)**			**Mode of conception**		**Age group (years)**			**Mode of conception**	
	**Up to 30**	**31 – 36**	**37 and older**	**SC**	**ARTC**	**Up to 30**	**31 – 36**	**37 and older**	**SC**	**ARTC**
	**n = 50**	**n =50**	**n = 26**	**n = 90**	**n = 36**	**n = 128**	**n = 168**	**n = 170**	**n = 205**	**n = 261**
Most antenatal care from specialist obstetrician in private consulting rooms	4.0	4.1	7.7	2.2	11.4	76.8	81.5	82.6	**70.3**	88.8***
**Consulted during pregnancy:**										
General practitioner	94.0	95.9	87.5	**97.8**	82.4*	81.7	73.5	77.3	**85.8**	70.0***
Hospital doctor	51.0	53.2	69.6	50.6	70.0	25.2**	**12.3**	22.7*	19.1	20.2
Non-obstetric medical specialist	16.7	20.9	19.0	14.3	32.1	15.3	**15.6**	24.8*	11.8	24.6
Midwife	85.1	95.7	95.7	90.6	93.8	46.4	45.6	47.8	**52.0**	42.1*
Physiotherapist / Chiropractor / Osteopath	17.0	22.7	37.5	22.9	25.0	**26.4**	40.8*	39.2*	32.7	38.9
Counsellor / Psychologist / Social Worker	8.5	15.9	22.7	14.5	13.3	5.6**	9.2*	**17.9**	8.2	13.9
Complementary health practitioner	17.5	28.2	38.1	26.8	24.1	24.5***	35.7*	**48.6**	33.5	40.5
Number of ultrasound scans Mean (SD)^1^	**2.62 (1.1)**	3.54 (2.8)	3.29 (1.6)*	**2.81 (1.4)**	3.82 (2.8)**	3.56 (2.1)***	3.65 (2.1)**	**4.66 (3.6)**	**3.31 (1.7)**	4.54 (3.3)***
Nuchal translucency test	71.4	86.0	79.2	78.4	80.0	**83.5**	92.5*	86.4	85.1	89.8
CVS or amniocentesis	16.0	18.0	15.4	17.8	13.9	14.8*	15.5*	**24.7**	19.5	18.0
Glucose Tolerance Test	89.8	95.9	95.5	90.9	100.0	97.7	95.1	97.0	96.1	96.9
24 hr Urine Collection	8.5	16.7	5.0	9.5	16.1	11.2	12.3	11.8	11.6	11.9
Cervical Suture	0.0	2.1	0.0	1.2	0.0	2.4	1.3	3.8	2.1	2.9
Overall antenatal care rated as good or very good	77.6	78.0	92.3	79.8	83.3	89.8	89.9	89.3	**83.9**	94.2***

^1^ For ease of interpretation, means are reported. However, non-parametric Mann–Whitney tests were used to test for between group differences, as the distribution was non-normal.

*p<0.05; **p<0.01; ***p<0.001.

Bold indicates statistically significantly different groups

#### Univariable comparisons of differences between women in each age group

*Physical health:* There were no significant differences between women in the three age groups in the incidence of most common, pregnancy-related health problems; overall pregnancy rated health; the SF12-PCS score or pregnancy-related hospital admissions. Women in the two older age groups were significantly more likely to report heart palpitations and placenta praevia than women in the youngest age group. Women in the oldest age group were significantly more likely to report pre-eclampsia than women in the youngest age group. Women in the oldest age group were significantly more likely to report feeling very well throughout the pregnancy than women in the middle age group.

*Mental health:* There were no significant age group differences in MCS or STAI State scores or self-reported experiences of intense anxiety or depression during pregnancy. Women in the older and middle age groups were more likely to report feeling calm and relaxed during pregnancy than women in the youngest age group. This overall appraisal was confirmed by the scores on the symptom measures. Women in the older age group had significantly lower scores on the EDS and the STAI Trait Inventory than women in the younger age group indicating that they were experiencing fewer symptoms of depression and that in general they responded to alarming situations with less anxiety.

*Health behaviours:* Older women were significantly more likely to report taking any prescription medicine during pregnancy than women in the other two age groups. There were no significant differences between women in the three age groups in self-reported use of over-the-counter medications, tobacco or alcohol. Older women were significantly less likely to participate in exercise at least weekly than women in the middle age group.

### Health service use

There were no significant differences between age groups in pregnancy-related hospital admissions.

*Women without private health insurance:* Women in the older age group reported having significantly more ultrasound scans than women in the youngest age group. There were no other significant age group differences in health providers who were consulted, tests and procedures undertaken or rating of antenatal care for women without private health insurance (see Table 3).

*Women with private health insurance:* Women in the oldest age group were significantly more likely to have consulted a non-obstetric medical specialist than women in the middle age group, and significantly more likely to have consulted mental health practitioners and complementary health practitioners than women in both the youngest and the middle age groups. They had more ultrasound scans than women in the youngest and middle age groups. Women in both the middle and the oldest age groups were more likely to have consulted a physiotherapist or related practitioner than women in the youngest age group. Women in the middle age group were significantly more likely to report having consulted a hospital doctor than women in the youngest and oldest groups, and were more likely to have had a nuchal translucency test than women in the youngest age group.There were no significant differences in the proportion reporting having had other tests and procedures, or in overall ratings of antenatal care (Table 3).

#### Univariable analysis: differences between SC and ARTC women

*Physical health:* There were no significant differences between SC and ARTC women in overall self-reported physical health, or in reporting of any incidence of most of the common physical health problems. ARTC women were however, significantly less likely to report back pain, stiff or painful joints and tender breasts than SC women. ARTC women were significantly more likely to report problems in placental functioning, vaginal bleeding and gestational diabetes than women who conceived spontaneously (see Table 2).

*Mental health:* There were no significant differences between ARTC and SC women in overall emotional health (MCS scores), self-reported anxiety and depression or proportion feeling calm and relaxed. However, ARTC women had significantly lower scores on the EDS, STAI state and STAI trait scores than SC women.

*Health behaviours:* There were no significant differences between ARTC and SC women with regard to self-reported smoking or drinking alcohol, use of prescription medications, or regular participation in exercise in pregnacy. ARTC were significantly more likely to report taking any over-the-counter medication than SC women.

### Health service use

Significantly more ARTC women had both pregnancy-related and non-pregnancy related hospital admissions than SC women.

*Women without private health insurance*: ARTC women were significantly less likely to report consulting general practitioners than SC women. ARTC women reported having significantly more scans than SC women. There were no significant differences between the two groups in other pregnancy procedures or tests, or in overall rating of antenatal care (see Table 3).

*Women with private health insurance*: ARTC women were significantly more likely to report that most of their antenatal care was provided by a specialist obstetrition in private consulting rooms than SC women. They were less likely to have consulted general practitioners and midwives than SC women. ARTC women reported having significantly more scans than SC women. There were no significant differences between the two groups as regards any of the other pregnancy procedures or tests. ARTC women were significantly more likely to rate their experiences of antenatal care as good or very good than SC women (Table 3).

#### Multivariable analyses

As there were significant differences between SC and ARTC groups in terms of some socio-demographic variables (see Table 1), country of birth, marital status and occupational status, and having private health insurance were included in the regression models. Language spoken at home was significantly associated with the outcome variables in univariate analysis, so this was also included. Demographic variables were entered first, then socio-economic indicators, then possible reproductive risk factors and, then, emotional health scores (model for PCS scores) or self-rated general health (model for MCS scores). The standardized coefficients for the two linear regression models are summarized in Table 4. When other factors were controlled speaking a language other than English at home, having private health insurance and expecting twins were associated with less optimal self-reported physical health. Controlling for other relevant factors, having private health insurance and having better physical health were associated with more optimal self-reported emotional health.

**Table 4:** Summary statistics for hierarchical regression for physical and mental component summary scores of the SF-12

		**Standardised coefficients: PCS score as outcome**				**Standardised coefficients: MCS score as outcome**			
	**Reference category**	**Step 1**	**Step 2**	**Step 3**	**Step 4**	**Step 1**	**Step 2**	**Step 3**	**Step 4**
Country of birth: Other	Australia	.022	.011	.001	.001	-.027	-.017	-.017	-.008
Language at home: Other	English only	-.108*	-.112*	-.117**	-.108*	-.032	-.022	-.022	.000
Marital status: Single or de facto	Married	.045	.019	.012	.024	-.107*	-.082	-.082	-.083
Occupational status: No paid employment	In paid employment		-.088*	-.061	-.060		.025	.025	.029
Health insurance: None	Yes		.102*	.088*	.097*		-.110*	-.110*	-.094*
Mode of conception: ARTC	SC			-.014	-.025			.025	.014
Age				.011	-.002			.041	.036
Expecting: Multiples	Singleton			-.197***	-.194***			.052	.050
Edinburgh Depression score					-.054				
Anxiety Trait score					-.113				
Self-rated general health (1=Poor to 5=Excellent)									.204***
**Adj R**^**2**^		**.080**	**.021**	**.055**	**.076**	**.008**	**.016**	**.017**	**.057**

MCS=Mental Component Summary; PCS=Physical Component Summary.

*p<0.05; **p<0.01; ***p<0.001.

In addition to the variables on which SC and ARTC women differed significantly, language spoken at home, PCS scores and STAI State Anxiety scores were included in the logistic regression model for any pregnancy-related hospital admission. Demographic variables were entered first, then socio-economic indicators, possible reproductive risk factors, physical health, and finally emotional health. When controlling for other factors (see Table 5), having a pregnancy related hospital admission was associated with having poorer self-rated physical health and expecting multiples.

**Table 5:** Summary statistics for logistic regression to identify factors associated with pregnancy-related hospital admissions

		**Step 1**	**Step 2**	**Step 3**	**Step 4**	**Step5**		
	**Reference category**	**Adj OR**	**Adj OR**	**Adj OR**	**Adj OR**	**Adj OR**	**95% C.I. for Adj OR**	
Country of birth: Other	Australia	0.525*	0.52*	0.569	0.567	0.567	0.312	1.032
Language at home: Other	English only	1.269	1.205	1.262	1.143	1.143	0.593	2.204
Marital status: Single or de facto	Married	1.008	1.01	1.064	1.058	1.058	0.585	1.913
Occupational status: No paid employment	In paid employment		1.742*	1.464	1.419	1.419	0.84	2.398
Health insurance: None	Yes		0.929	0.772	0.719	0.729	0.397	1.337
Mode of conception: ARTC	SC			1.605	1.662	1.662	0.977	2.827
Age group: 31-36	Up to 30			1.068	1.041	1.041	0.572	1.894
Age group: 37 and older	Up to 30			1.069	1.091	1.091	0.579	2.057
Expecting: Multiples	Singleton			3.159**	2.502*	2.502*	1.068	5.861
PCS score					0.971*	0.971*	0.945	0.998
Anxiety State score					1.016	1.016	0.989	1.044
**Nagelkerke R**^**2**^		**0.016**	**0.03**	**0.064**	**0.079**	**0.083**		

Adj OR=Adjusted Odds Ratios; C.I.=Confidence Interval; PCS=Physical Component Summary.

*p<0.05; **p<0.01; ***p<0.001.

## Discussion

This study has considerable strengths in recruiting a stratified sample of sufficient size and collecting data about relevant potential confounding factors to elucidate the interactions among mode of conception, age, health service use, health behaviours and women’s health in pregnancy. We acknowledge a number of limitations. First that socioeconomically advantaged women were over-represented in the sample and that the findings might not reflect the situation of women in a lower socioeconomic position. Second that the data are self-report and were not confirmed from medical records. Third a few questionnaire items used technical terms that were not widely understood and yielded uninterpretable data. For example we asked about pre-implantation genetic diagnosis which can only be conducted prior to assisted conception, but some women who conceived spontaneously reported that they had undergone this procedure suggesting that the term was not meaningful and we have therefore not reported it.

Overall physical health assessed by the SF12 Physical Component Score in this cohort was lower in all groups than that reported for the randomly selected sample of women in the Australian Longitudinal Study of Women’s Health’s (ALSWH) young (18 – 22) (PCS 48.89 ± 8.70) and mid aged (45 – 49) (PCS 49.55 ± 9.32) cohorts [36]. This suggests that advanced pregnancy itself, regardless of age or mode of conception imposes physical demands which reduce overall functioning. This was reflected in the finding that only about a third of women reported feeling overall very well through pregnancy. It was striking that apart from pre-eclampsia and placenta praevia, most physical symptoms were not more common among older than younger women. In the absence of other symptom differences, the meaning of the lower rates of self-reported heart palpitations among younger women are unclear, but might reflect differences in intrinsic fitness. Our data confirmed the existing evidence [8,9] that women conceiving with ART were more likely than those conceiving spontaneously to experience gestational diabetes, vaginal bleeding and placenta praevia. However, fewer ARTC than SC women reported non-specific symptoms like back pain, joint pain and breast tenderness suggesting that perception of these might to some extent be governed by sense of entitlement to complain, which might be lower among women whose conceptions have required major technical interventions and financial costs. Multivariable analysis revealed however that neither maternal age nor mode of conception was associated directly with PCS scores. Indirectly, worse physical health was associated with multiple gestation which was more common among ARTC than SC women and among those with private health insurance which is much more commonly held by older than younger women. Speaking a language other than English at home is an indicator in Australia of lower socioeconomic status and in this circumstance might also reflect greater difficulty in negotiating to have health care needs met adequately by providers.

In contrast, overall mental health assessed by the SF12 Mental Component Score was higher in all groups than in the ALSWH young (MCS 45.35 ± 12.36) and mid aged (MCS 46.71 ± 12.29) cohorts [36]. The mean EPDS scores in all groups were lower than the mean of 6.7 (± 4.9) at 32 weeks gestation reported in the Avon Longitudinal Study of Parents and Children [37] community cohort. More than half reported feeling mostly calm and relaxed. This indicates that the PATPA cohort in general had good mental health, with few limitations on their functioning related to mental health problems and probably reflects that they were a generally socioeconomically advantaged cohort [38]. The high rates of self-reported intense anxiety in early pregnancy support the existing evidence that pregnancy-specific anxiety is high in most women in the first trimester, but does not meet criteria for an anxiety disorder. There were few age related differences, but in general mental health appeared to be better in older than younger women. Older women had significantly lower levels of depressive symptoms and higher rates (almost 70%) of feeling mostly calm and relaxed. They had lower trait anxiety suggesting that greater life experience might contribute to more effective management of anxiety-arousing situations including during pregnancy. Confirming previous findings ARTC women had lower levels of symptoms of depression and anxiety than SC women and very few scored above the EPDS clinical cut-off score. In multivariable analyses neither age nor mode of conception were associated significantly with MCS scores. Overall women with better physical health and who were more affluent as indicated by holding private health insurance had better mental health.

Overall this cohort had low rates of smoking and most were not drinking any alcohol and were exercising regularly during pregnancy. There were very few differences in health behaviours between groups. Older women were less likely to participate in regular exercise and more likely to report using prescription medication than other age groups, which might reflect higher rates of coincidental chronic health conditions. There were high rates of use of over-the-counter medications, predominantly nutritional supplements, but ARTC women more likely to be doing this than SC women perhaps reflecting high consciousness of foetal health.

Our data suggest that the use of health services is governed primarily by affordability and capacity to pay. For women receiving fee-free health care in the public sector there were very few differences between age or mode of conception groups. The only differences were that ARTC were less likely to consult general practitioners and had more ultrasound scans than SC women. This suggests that they were more likely to be receiving the specialist antenatal care provided in tertiary hospitals than the antenatal care that is provided to women at low obstetric risk in the community by general practitioners. The only between age group difference was that older women had more ultrasound scans than the youngest women which are probably associated with prenatal genetic screening and diagnostic testing.

There were more differences between the groups among those with private health insurance. Private health insurance contributes to the costs of allied health services, and is also an indicator of higher socioeconomic status and capacity to pay for discretionary health care. In this group older women more likely to have consulted allied and complementary health services than other insured women. Older women reported no more musculoskeletal problems in pregnancy than the two younger age groups, but were more likely to have consulted physiotherapists and chiropractors. Similarly, although they reported fewer mental health problems they were more likely to have consulted an allied mental health professional. They also had more ultrasound scans and were more likely to have consulted non-obstetric medical specialists than younger insured women. ARTC women with private health insurance were significantly more likely than insured SC women to use private obstetricians rather than GPs or midwives, for antenatal care. They had more ultrasound scans and were more likely to rate their antenatal care in general as good or very good than SC women.

Neither age nor mode of conception was associated directly with pregnancy related hospital admission. The main contributor to antenatal hospital admission was multiple gestation almost all of which was among ARTC women and, not surprisingly it was also associated with self-reported poorer physical health.

## Conclusion

Overall these data suggest that in a high-income country with sophisticated and accessible health services, pregnancy health and health service use are governed by socioeconomic factors to a greater extent than they are by age or mode of conception. While rates of some pregnancy health problems increase with age the adverse consequences of these for self-rated physical health appear to be offset by having access to high quality multi-disciplinary health care. Pregnancy mental health appears to be better among the most advantaged women who have greater capacity to access health services, but probably also greater personal confidence and maturity and more secure economic circumstances and housing. Pregnancy-related hospital admission rates are likely to improve with the practice of transferring a single embryo during assisted conception and thereby reducing the multiple gestation rates. These data confirm that in general ARTC women are delighted to be pregnant, but perhaps idealize motherhood and are insufficiently prepared for the inevitable losses and adjustments that it involves.

## Competing interest

The authors declare that they have no conflicts of interest.

## Authors’ contributions

CM, JF, KH, FG, JB and JMcB conceptualized and designed the study and collected the data. KW, JF and KH undertook data analysis and interpretation. JF with KW and KH wrote the first draft of the manuscript. All authors approved the final draft.

## Pre-publication history

The pre-publication history for this paper can be accessed here:

http://www.biomedcentral.com/1471-2393/13/88/prepub
